# A Low-Cost Multibeam Switching Antenna Using Reconfigurable Hybrid Metasurface for Beamforming Applications

**DOI:** 10.3390/mi14081631

**Published:** 2023-08-18

**Authors:** Lili Sheng, Yumei Luo, Gangxin Ning, Liang Meng, Weiping Cao

**Affiliations:** 1 Guangxi Key Laboratory of Wireless Wideband Communication and Signal Processing, Guilin University of Electronic Technology, Guilin 541004, China; 2 School of Electronic Information and Automation, Guilin University of Aerospace Technology, Guilin 541004, China; 3 Guangxi Key Laboratory of Information Materials, Guilin University of Electronic Technology, Guilin 541004, China; 4 The 34th Research Institute of China Electronics Technology Group Corporation, Guilin 541010, China

**Keywords:** multibeam antenna, hybrid metasurface, beamforming, low cost, compact structure

## Abstract

In this paper, we proposed a multibeam switching antenna based on a low-cost reconfigurable hybrid metasurface applied for beamforming systems. The antenna consists of two parts: a microstrip feed antenna and a transmission hybrid metasurface. The latter is composed of three types of units with different amplitude and phase responses to electromagnetic waves so as to control the beams of the feed antenna. Sixteen PIN diodes are arranged in the metasurface with a simple bias network. When two different direct-current voltages are applied to the PIN diodes, the antenna can dynamically switch between two beams and four beams. For demonstration, the proposed antenna is fabricated, and the measured results show that the antenna operates at 9.07–9.42 GHz (−10 dB bandwidth) with a total size of 1.80λ_0_ × 1.52λ_0_ × 0.22λ_0_ (λ_0_ corresponds to the wavelength of 9.28 GHz in free space). With the merits of a compact structure, low cost and good radiation performance, the proposed design is suitable for beamforming applications.

## 1. Introduction

The multibeam antenna can accurately point to multiple users in time and has the advantages of multi-dimensional coverage and a fast communication rate, so it is of great significance in beamforming applications. With the rapid development of the low-orbit satellite communication industry and 5G wireless communication, the Internet-of-Space-Things (IoST) system [1], mainly composed of ground stations, aerial vehicles and small satellites, has attracted more and more interest. The antennas used in the IoST system should provide the best possible performance and maintain the minimum size. The dynamical multibeam switching antenna (MSA) has become a potential candidate for IoST systems due to its ability to cover multiple areas, improve multipath effect and support high data rates with a compact structure [2]. The flat MSA can also be widely applied in and beyond 5G and 6G mobile communication systems and multi-target radar systems [3].

Generally, the categories of MSA mainly include phased array antenna [2,4], multi-port antenna [5,6] and coding metasurface antenna [7,8]. As mentioned in [9], the phased array antennas can achieve flexible beam switching, but the configuration is complicated with a complex structure, and they suffer high costs. Compared with digital beamforming methods, MSAs based on metasurfaces have lower energy consumption and complexity. As discussed in [10], four ports in each unit are used to generate various radiation beams. However, when a large-scale antenna array is composed of these units, the feed network of the system is quite complex. In recent years, with the introduction of digital coding metasurfaces [11], there are more choices for the design of an MSA. Active devices, such as a PIN diode or varactor, are added to the coding metasurface to discretize the phase or amplitude response of the electromagnetic wave so as to obtain flexible beam control capability. For example, a 2-bit programmable metasurface in [12] is adopted to realize flexible multibeam switching. However, these reflective or transmissive metasurfaces require additional horns to irradiate, resulting in a bulky system volume. In addition, as discussed in [13], a reconfigurable metasurface loaded with PIN diodes is designed to achieve beam switching. However, this method uses a large number of PIN diodes, which undoubtedly increases the loss and cost. Of course, the method of mechanically rotating the coding metasurface can also achieve multibeam switching [14]. However, it lacks real-time capability and flexibility with heavy weights.

The hybrid metasurface is composed of units with different amplitudes and phase responses to incident waves, which makes the manipulation of electromagnetic waves more flexible. For compact and low-cost multibeam antennas based on metasurfaces, hybrid metasurfaces are a promising choice. As presented in [15], a hybrid metasurface with a split-ring resonator and U-shaped unit cell is adopted to split a beam. A hybrid digital coding metasurface consisting of a series of I-shaped structures was proposed in [16] to independently manipulate surface waves and spatially propagating waves. However, once this type of metasurface is fabricated, the generated beams are typically static and cannot be adjusted in real time. In the case of beamforming, it is necessary to steer the pattern radiated by the antenna in real time; obviously, the antenna with a fixed beam cannot achieve this.

In this paper, a single-layer reconfigurable hybrid metasurface is innovatively proposed for the realization of a compact, real-time and low-cost MSA. Firstly, in order to realize the compact structure, a planar microstrip feed antenna is designed. Secondly, three kinds of metasurface elements with different phase and amplitude responses to electromagnetic waves are proposed and studied. Then, the three elements are combined to form different hybrid metasurfaces. Under the irradiation of the feed antenna, the manipulation of the far-field radiation beam using the hybrid metasurface is studied. Finally, after optimization, by adding sixteen PIN diodes in the hybrid metasurface, real-time switching between two beams and four beams can be achieved. The proposed multibeam switching antenna features flexible beam switching, a small size and a simple feeding network with low costs. The idea of hybrid units can be applied to the design of a large aperture metasurface.

## 2. Antenna Design

### 2.1. Design of the Feed Antenna

**Figure 1:**
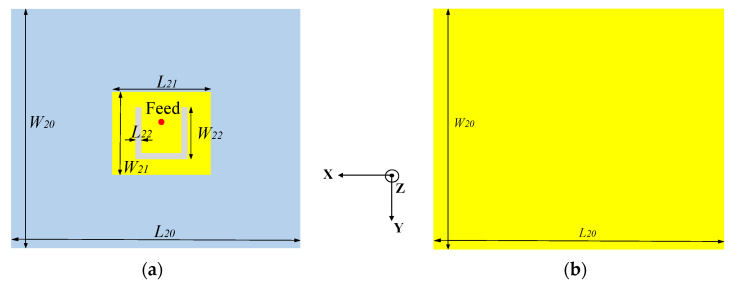
The structure of the feed antenna. (**a**) Front view; (**b**) back view.

**Figure 2:**
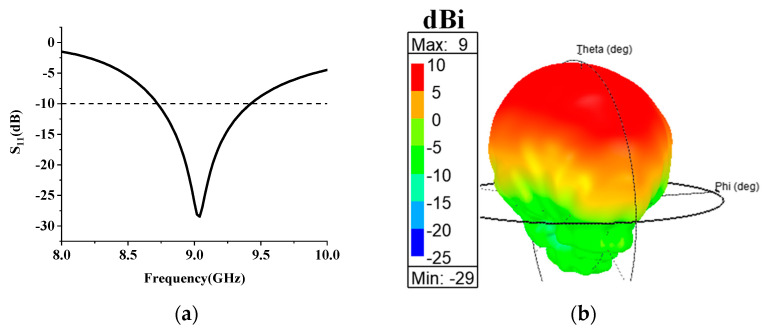
The simulated results of the feed antenna. (**a**) S_11_; (**b**) 3-D gain pattern.

In order to realize the low-profile characteristic of the antenna, a planar microstrip antenna is adopted instead of the horn as the metasurface feed. Figure 1a shows the front structure of the feed antenna, with the yellow part being copper and the gray part being a 1.5 mm thick F4B dielectric substrate ($\varepsilon_{r}=2.2$; tanδ=0.001). A U-shaped slot is etched on the radiation patch to improve the current distribution and achieve a wider bandwidth and higher gain than an ordinary microstrip without a slot. The back of the substrate is covered with copper to ensure that the antenna keeps the broadside radiation mode, as shown in Figure 1b. It can be seen from Figure 2a that the antenna operates in the range of 8.7–9.5 GHz, and Figure 2b shows the 3D pattern of the antenna with a peak gain of 9 dBi. 

### 2.2. Unit Design of the Hybrid Metasurface

**Figure 3:**
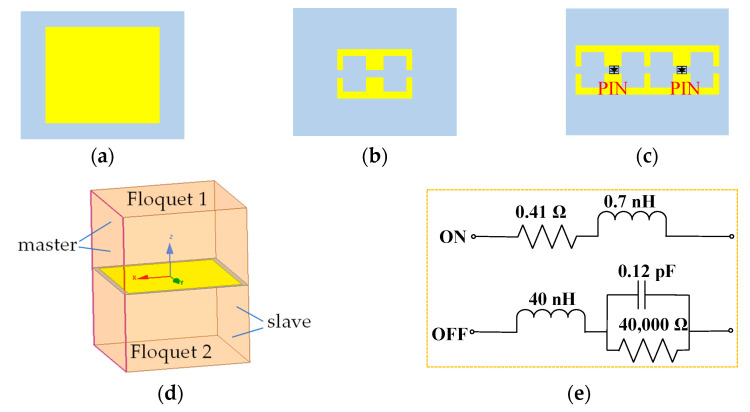
Three types of units. (**a**) unit 1; (**b**) unit 2; (**c**) unit 3; (**d**) diagram of unit cell boundary conditions; (**e**) the equivalent circuit of PIN diode.

The unit cells of the proposed hybrid metasurface are illustrated in Figure 3a–c. F4B (marked gray) with a dielectric constant of 2.2, a loss tangent of 0.001, and a thickness of 1 mm is used as the dielectric substrate. The thickness of the metal copper (marked yellow) is 0.035 mm. Two types of radiation structures are selected: a rectangular patch (unit 1) and two electric-inductive–capacitive (ELC) resonator structures (unit 2 and unit 3). Unit 1 is a common structure that makes up a metasurface, and different phase responses to transmitted electromagnetic waves can be achieved by adjusting the size of the rectangular patch. Unit 2 has resonance characteristics at a certain frequency bandwidth, and if it is considered as a component of the metasurface, it has filtering characteristics for specific polarized electromagnetic waves during resonance [17]. By combining the above two units into a metasurface, the phase and amplitude of the transmitted beam can be adjusted simultaneously. Unit 3 has made the following improvements on the basis of unit 2: two structures of unit 2 are merged to achieve a more compact metasurface, and diodes are loaded on the two wide arms in the middle to achieve real-time manipulation of electromagnetic waves. A commercially available “SMP 1320-079LF” is chosen as the switchable diode as it has low resistance and low capacitance. According to the datasheet [18], the forward voltage of the diode is 0.85 V, and for the convenience of testing, a 1.5 V battery is used for DC bias. We define the state of the diode as “ON” when 1.5 V voltage is loaded; when 0 V voltage is loaded, the state is defined as “OFF”.

**Figure 4:**
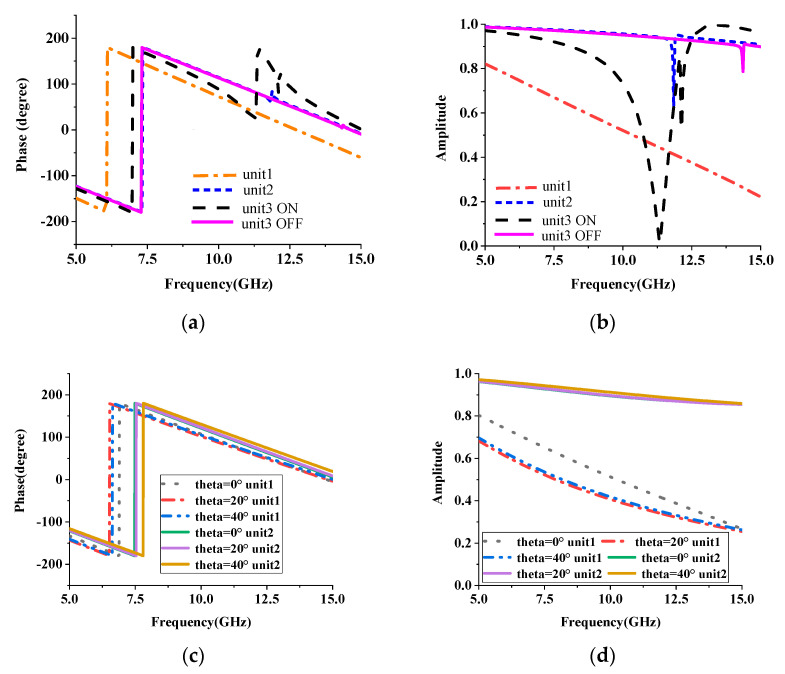
Transmission characteristics of three types of units. (**a**) Phase of different units; (**b**) amplitude of different units; (**c**) phase at different incident angles; (**d**) amplitude at different incident angles.

The transmission characteristics are simulated using ANSYS Electromagnetics Suite. Each unit is simulated using periodic boundary conditions and Floquet ports, as shown in Figure 3d. The incident uniform plane wave is a TE linearly polarized wave. During the simulation, we used the equivalent circuit shown in Figure 3e to replace the OFF or ON state of the diode. From Figure 4a,b, we know that unit 1 and unit 2 have different amplitude and phase responses to electromagnetic waves, enabling the manipulation of electromagnetic waves from two dimensions. Unit 2 and unit 3 (in OFF state) have almost the same amplitude and phase properties at 11.2 GHz. In terms of amplitude, when all PIN diodes are in the ON state, the transmissivity of the TE wave in unit 3 is almost 0 at 11.2 GHz. When all PIN diodes are in the OFF state, the transmissivity is 0.96. When electromagnetic waves are incident from different angles, the phase and amplitude responses of the units remain relatively stable. It should be noted that due to the similar characteristics of unit 3 and unit 2, Figure 4c,d only show the situation of unit 1 and unit 2. The amplitude difference close to 1 will have a great impact on electromagnetic waves and help the metasurface to manipulate the transmitted waves.

### 2.3. Design and Optimization of Multibeam Metasurface Antenna

It can be seen from Section 2.2 that there are phase differences among the three types of unit cells, and they have different transmittances. Therefore, by arranging these three units, a metasurface with an amplitude and phase gradient can be formed to control the antenna radiation beam. The effect of the metasurface with different combinations on the far-field radiation pattern is studied by placing the metasurface at the same height above the feed antenna and keeping other conditions unchanged.

**Figure 5:**
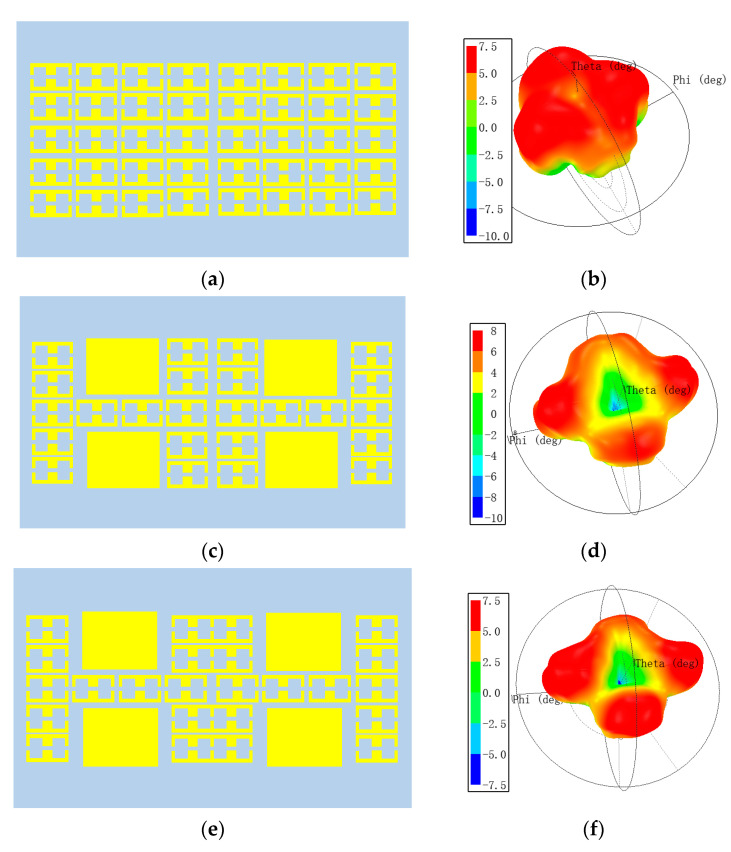
Different combinations of the hybrid metasurface and corresponding far-field patterns at 9.28 GHz. (**a**) case 1; (**b**) the far-field pattern of case 1; (**c**) case 2; (**d**) the far-field pattern of case 2; (**e**) case 3; (**f**) the far-field pattern of case 3.

As shown in Figure 5a,b, when the metasurface only includes unit 2, it can be seen that the antenna only radiates one beam and has strong scattering. When the metasurface is combined with units 1 and 2, as shown in Figure 5c,d, the antenna exhibits a zero point in the theta = 0 direction, and the directionality is relatively enhanced compared with case 1. As shown in Figure 5e,f, after replacing unit 2 in the middle with unit 3, the beam radiated by the antenna is more symmetrical compared with that in case 2. From the above three cases, it can be seen that when the hybrid metasurface consists of different units and is irradiated with the planar feed, it tends to realize multiple beams due to the difference in phase and amplitude.

**Figure 6:**
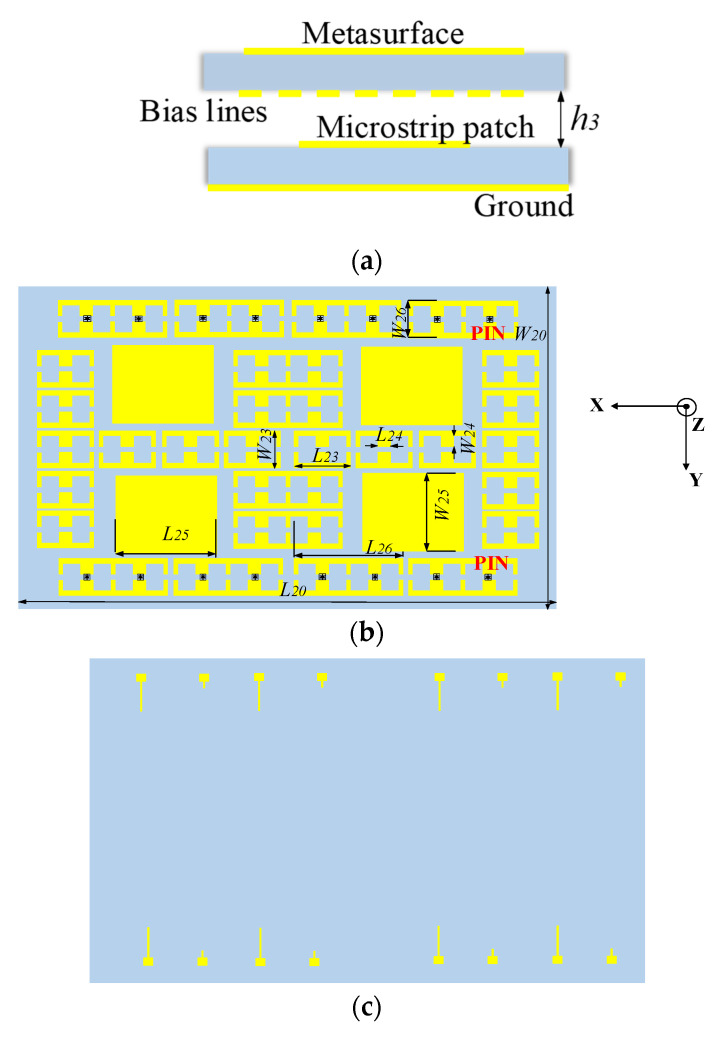
The structure of the metasurface. (**a**) Side view of metasurface antenna; (**b**) front view of metasurface; (**c**) bottom view of metasurface.

After heuristic optimization, the metasurface antenna as shown in Figure 6 is finally obtained. The proposed switched-beam antenna consists of two layers, as shown in Figure 6a. The bottom layer is a microstrip antenna (see Figure 1), and the upper layer is the hybrid metasurface. Eight PIN diodes are placed in the cells of the first row of the hybrid metasurface. And the other eight PIN diodes are placed in the last row with simple bias lines on the back (see Figure 6). The bias lines in Figure 6c are connected to the units with PIN diodes on the front side through a metal via. The shorter wire is connected to the positive pole of the DC circuit. To reduce the impact of DC bias on microwave signals, the width of the bias line is only 0.2 mm for high impedance. At the same time, in order to prevent microwave signals from entering the DC path, the bias line is connected to the corner with the lowest voltage in each unit. In order to be as realistic as possible, the bias lines and the metal vias are taken into the simulation.

**Figure 7:**
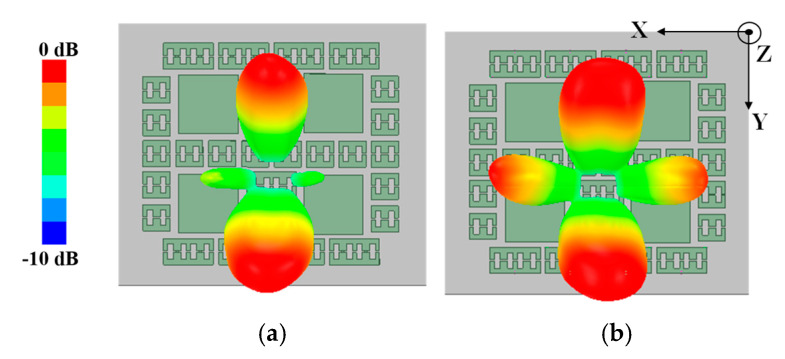
Simulated 3-D normalized gain patterns with all PIN diodes in (**a**) ON state and (**b**) OFF state.

The two layers are separated by an air cavity with the height of *h*_3_. As shown in Figure 7, when all PIN diodes are turned on, the antenna radiates two beams; when all PIN diodes are in the OFF state, the antenna radiates four beams. It must be noted that the patterns in Figure 7 are observed at 9.28 GHz, which is lower than the results discussed in Figure 4. This is due to the non-periodic arrangement of the units, which is different from Figure 3. Another reason is the difference in incident plane waves. The simulation of the unit uses uniform plane wave incidence, while the proposed metasurface is illuminated using electromagnetic waves radiated by a planar microstrip feed. These reasons result in a red shift in the operating frequency of the antenna.

**Figure 8:**
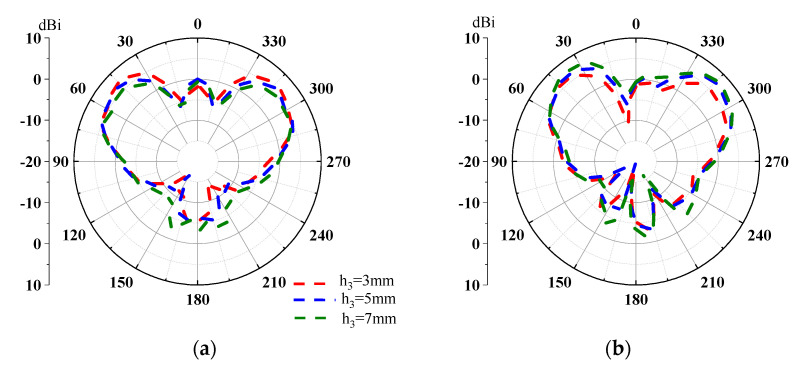
Two-dimensional radiation pattern of antenna with different h_3_ values. (**a**) XOZ-plane; (**b**) YOZ-plane.

In order to obtain better radiation performance, the parameter *h*_3_ is analyzed. The boundary between the induction near-field and Fresnel region is taken as the original value of *h_3_*, which is calculated as (1).

(1) $$h_{3}\leq 0.62\sqrt{L^{3}/\lambda }$$

where *L* is the effective length of the source antenna, and *λ* is the wavelength in free space at 9.28 GHz. According to Equation (1), the initial value of *h*_3_ is 7.4 mm. In order to reduce the antenna profile while maintaining good impedance matching and a low side lobe, the radiation patterns with *h*_3_ of 3 mm, 5 mm and 7 mm are analyzed. Figure 8 shows the two-dimensional pattern of the antenna when all diodes are OFF. When *h*_3_ is equal to 3 mm, as shown with the red curve in Figure 8, the gain of the antenna is lower in the YOZ-plane. When *h*_3_ is equal to 7 mm, as shown with the green curve in the XOZ-plane, the larger tail lobe of the antenna results in a lower gain. Overall, the antenna radiation performance is the best when *h_3_* is equal to 5 mm. Table 1 shows the final parameter values after optimization.

### 2.4. Mechanism of Multibeam Switching Antenna Based on the Reconfigurable Hybrid Metasurface

As shown in Figure 4, the hybrid metasurface consists of three types of units that have different transmission characteristics. We study it from the aspect of phase and amplitude, respectively. From the perspective of phase, according to the generalized Snell’s law [19], i.e., Equation (1) below, when there is a phase gradient *dφ*/*dx*, the incident electromagnetic wave will be transferred to anomalous refraction. When the incident angle *θ_i_* is known, the refraction angle *θ_t_* can be designed using the phase gradient *dφ/dx* caused by the interface.

(2) $$n_{t}sin\theta_{t}-n_{i}sin\theta_{i}=\frac{\lambda_{0}}{2\pi }\frac{d\varphi }{dx}$$

where *n_i_* and *n_t_* are the refractive indices of the two media. For the reconfigurable hybrid metasurface, the units are non-periodically arranged, and the phase gradient introduced is not continuous. From this analysis, we know that the proposed hybrid metasurface can undergo abnormal refraction of incident electromagnetic waves.

In terms of amplitude, when all PIN diodes are in the ON state, the transmissivity of the TE wave in Unit 3 is almost 0. This means that when the electromagnetic wave irradiates the unit with low transmittance, the electromagnetic wave will be reflected back for secondary radiation. According to Huygens’s principle, the radiated electromagnetic wave will be continuously superimposed to form a new radiation pattern. The amplitude difference and phase shift can be used to control the direction and number of the radiation beams. Generally, the transmission wave from the hybrid metasurface under plane-wave illumination can be calculated as the following equation [20]:

(3) $$f\left(\phi ,\theta \right)=\sum_{m=1}^{M}\sum_{n=1}^{N}A(m,n)e^{-j\varphi (m,n)}e^{-jkdsin\theta [\left(m-\frac{1}{2}\right)cos\phi +(n-\frac{1}{2})sin\phi )]}$$

where *A*(*m, n*) and *φ*(*m, n*) denote the transmission amplitude and phase from the *(m, n)* unit cell, d is the periodicity of the unit and (*ϕ, θ*) represent the azimuth and elevation angles of the radiation pattern, respectively.

**Figure 9:**
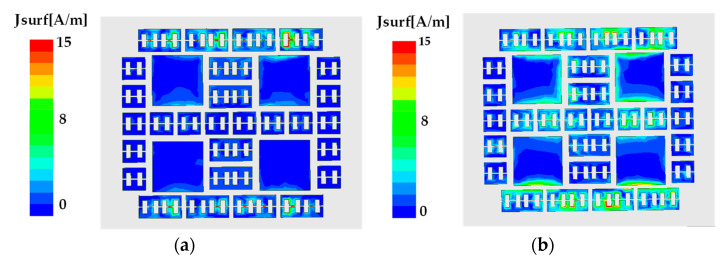
Current distribution in the hybrid metasurface. (**a**) ON state; (**b**) OFF state.

To better reveal the radiation properties of the multibeam switching antenna, the current distribution on the hybrid metasurface is studied as shown in Figure 9. It can be seen that when all PIN diodes are in the ON state, the current density of unit 1 and unit 2 is low, while the current distribution on unit 3 located on both sides is large. At this time, two beams in the YOZ-plane were obtained. When all PIN diodes are in the OFF state, the current density of unit 2 and unit 1 in the middle row obviously increases, while the current on both sides remains large. In this case, the antenna not only maintains two radiation beams on the YOZ-plane but also radiates two beams on the XOZ-plane.

## 3. Experiments and Results

**Figure 10:**
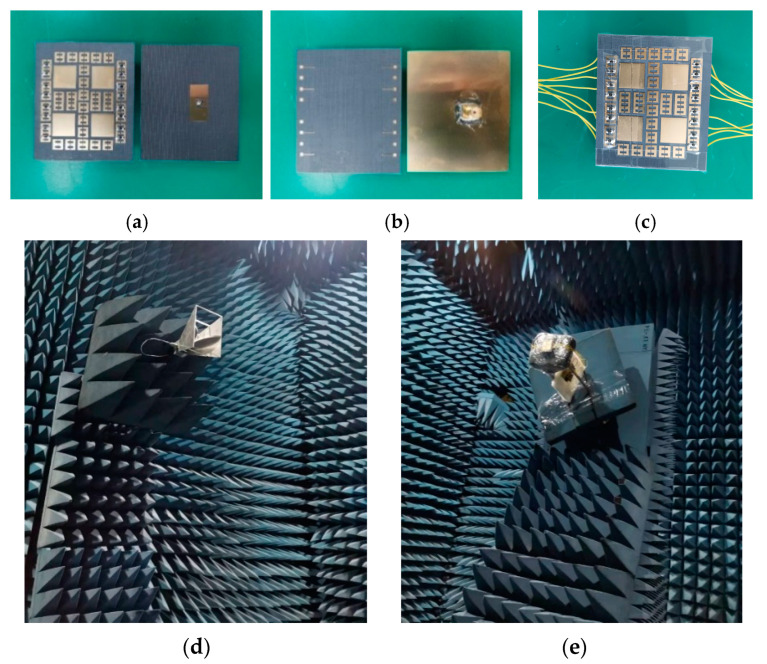
Fabricated prototypes and testing environment. (**a**) Front view and (**b**) back view of the hybrid metasurface and the source antenna; (**c**) front view of overall structure; (**d**) standard receiving antenna in microwave anechoic chamber; (**e**) the proposed antenna under test.

**Figure 11:**
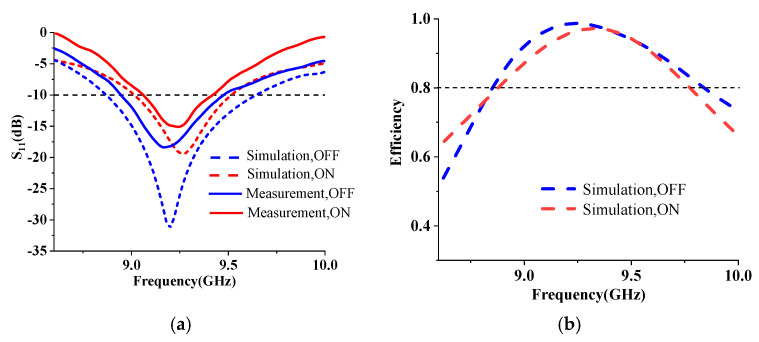
Measured and simulated results. (**a**) Reflection coefficient; (**b**) simulated efficiency.

As illustrated in Figure 10, the hybrid metasurface antenna was fabricated and tested. The total dimension is 58 mm × 49 mm × 7 mm (1.80λ_0_ × 1.52λ_0_ × 0.22λ_0_). The bias lines are placed on the back of the metasurface through the vias. Figure 11a presents the comparison of the reflection coefficient between measurement and simulation, indicating that the measured −10 dB impedance bandwidth is 9.07–9.42 GHz when all PIN diodes are in the ON state. The measured bandwidth is narrower than the simulation because of manufacturing errors.

Figure 11b gives the simulated radiation efficiency, from which it can be seen that in the working band of the antenna, the radiation efficiency under two states is greater than 80%. Although the amplitude is modulated by the proposed metasurface, the total radiation efficiency of the antenna is not affected since the transmission amplitude of unit 2 and unit 3 (in the OFF state) is close to 1.

**Figure 12:**
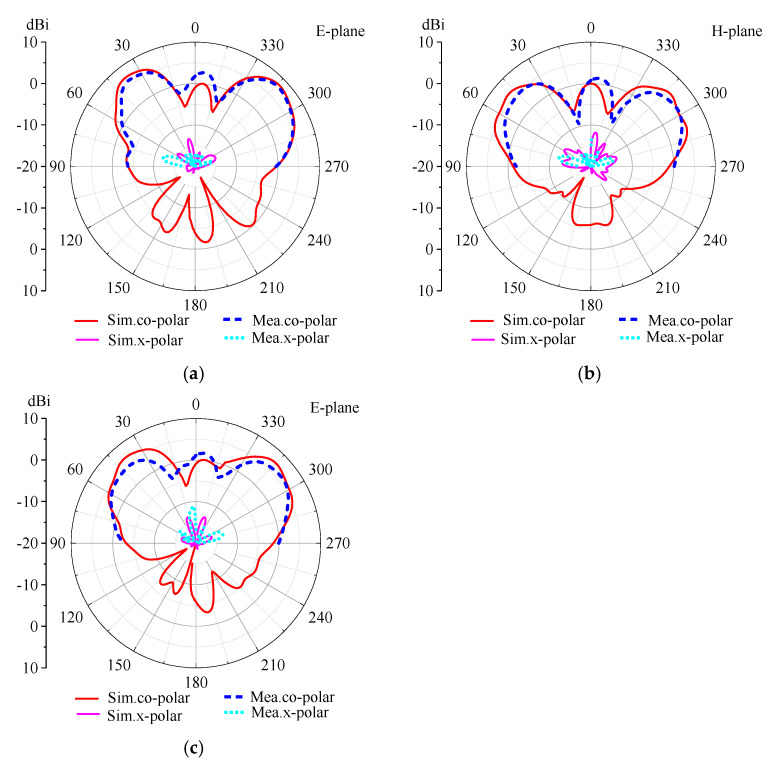
The realized gain patterns. (**a**) Two beams in E-plane; (**b**) four beams in H-plane and (**c**) E-plane.

Figure 12 shows the 2-D realized gain patterns at 9.28 GHz. It is worth noting that only the patterns in upper-half-plane were measured for their directional radiation properties. When all PIN diodes are in the ON state, the antenna radiates two beams in the E-plane, which is located at theta = 38°, 314° in polar coordinate with the measured gain of 6.77 dBi and 7.32 dBi, respectively. When all PIN diodes are in the OFF state, the antenna generates four beams, two of which are located in the H-plane with theta = 48°, 312°, and the other two are in the E-plane with theta = 42°, 314°. The measured gains of the four beams are 5.54 dBi and 5.37 dBi at the H-plane and 5.76 dBi and 5.98 dBi at the E-plane, respectively. The measured gain of cross-polarization is lower than −12 dBi. It can be seen that the measured gain is lower than the simulation, and the side lobe is higher than the simulation results. This is mainly caused by the inserted loss of PIN diodes and the influence of the bias lines. Nevertheless, the measured results are basically consistent with the simulation results, indicating that the proposed method based on a reconfigurable hybrid metasurface is feasible.

## 4. Performance Comparison

To further illustrate the advantages of the proposed multibeam switching antenna, the comparisons with the multibeam antennas based on metasurfaces are listed in Table 2. The antenna presented in [15] with a smaller size achieves two beams of radiation, but it cannot switch beams. Antennas using multiple ports to achieve multiple beams were proposed [21,22,23,24], but they can only achieve one beam at the same time, and the real-time switching of different ports is relatively complex. The antenna proposed in [25] achieved flexible beam control by placing programmable metasurface elements in SIW antennas, but the size of the antenna is relatively large. And as the array expands, the bias network of PIN diodes becomes complex. Our proposed multibeam switching antenna features flexible beam switching, a small size and a simple feeding network with a low cost. The idea of hybrid units can be applied to the design of a large aperture metasurface.

## 5. Conclusions

A novel reconfigurable hybrid metasurface antenna achieving multibeam switching is presented in this paper. As an elaborate arrangement of three types of metasurface elements, fewer PIN diodes are used, and the bias network is simple. The measured results are consistent with simulations, indicating that the antenna can realize dynamic switching between two beams and four beams with a compact structure. The proposed method is suggested for beamforming systems. And the possibility of flexible real-time control of more beams in a broad bandwidth will be investigated in future work.

## Figures and Tables

**Table 1:** Parameters of the proposed antenna (unit: mm).

***L*_20_**	***L*_21_**	***L*_22_**	***L*_23_**	***L*_24_**	***L*_25_**	***L*_26_**
58	16.7	0.38	4.7	1.5	8.5	8.7
***W*_20_**	***W*_21_**	***W*_22_**	***W*_23_**	***W*_24_**	***W*_25_**	***W*_26_**
49	8.1	6.28	4.5	1	9	5

**Table 2:** Comparison of proposed works with other reported metasurfaces.

Ref.	Operating Band	Overall Dimensions (*λ*_0_^3^)	Components	Beam Number	Aim	Complexity
[15]	3.4–3.6 GHz	0.61 × 0.47 × 0.02	A hybrid metasurface layer and a feeding antenna	2	Beam split	normal
[21]	5.2–5.5 GHz	2.23 × 1.85 × 0.06	A metasurface layer and a two-port feeding network	2	Dual-beam steering	normal
[22]	3.3–3.6 GHz	0.75 × 2.22 × 0.09	A metasurface layer and a two-port feeding network	4	Dual-beam steering	normal
[23]	5.6–5.9 GHz	2.52 × 2.52 × 0.15	Two metasurface layers and a four-port feeding antenna array	4	Beam steering	complex
[26]	5.5–6.2 GHz	4.25 × 4.25 × 1.95	Four metasurface layers and a feeding antenna	4	Obtain quad-beam	complex
[24]	2.1–3.5 GHz	1.12 × 1.12 × 0.10	Two metasurface layers and a four-port feeding antenna	4	Generate four beams	complex
[25]	11.6–12.2 GHz	4.80 × 0.64 × 0.02	Programmable metasurface with 16 PINs on an SIW antenna	1/2/3/4	Generate multibeam or directive beams	normal
This work	9.07–9.42 GHz	1.80 × 1.52 × 0.22	A hybrid metasurface layer with 16 PINs and a feeding antenna	2/4	Multibeam switching	easy

## Data Availability

Data are available based upon reasonable request from the corresponding author.

## References

[1] Kamil T., Weronika K., Mateusz R., Lukasz K., Krzysztof N. (2022). Multibeam Antenna for Ka-Band CubeSat Connectivity Using 3-D Printed Lens and Antenna Array. IEEE Antennas Wirel. Propag. Lett..

[2] Hong W., Jiang Z.H., Chao Y., Zhou J.Y., Chen P., Yu Z.Q., Zhang H., Yang B.Q., Pang X.D., Jiang M. (2017). Multibeam Antenna Technologies for 5G Wireless Communications. IEEE Trans. Antennas Propag..

[3] Dicandia F.A., Fonseca N.J.G., Bacco M., Mugnaini S., Genovesi S. (2022). Space-Air-Ground Integrated 6G Wireless Communication Networks: A Review of Antenna Technologies and Application Scenarios. Sensors.

[4] Cheng X., Zhang Q., Yao Y., Wang C., Yu T., Yu J., Chen X. (2022). W-Band Binary Phase-Controlled Multibeam Antenna Array Based on Gap Waveguide Magic-Tee. IEEE Trans. Antennas Propag..

[5] Luo J., Li L., Su J.R., Ma R.B., Han G.R., Zhang W.M. (2021). Multibeam Antenna Based on Partially Reflecting Defected Metasurface. IEEE Antennas Wirel. Propag. Lett..

[6] Sultan K., Ikram M., Trong N.N. (2022). A Multiband Multibeam Antenna for Sub-6 GHz and mm-Wave 5G Applications. IEEE Antennas Wirel. Propag. Lett..

[7] Ebrahimzadeh R., Zakeri B., Darvish A., Hosseininejad S.E. (2022). Multi beam scanning programmable metasurface using miniaturized unit cells for 5G applications. J. Electromagn. Waves Appl..

[8] Yu J.Y., Zheng Q.R., Zhang B., He J., Hu X.M., Liu J. (2022). Real-time programmable coding metasurface antenna for multibeam switching and scanning. Chin. Phys. B.

[9] Zhu Y., Deng C. (2022). Millimeter-Wave Dual-Polarized Multibeam Endfire Antenna Array with a Small Ground Clearance. IEEE Trans. Antennas Propag..

[10] Wu J., Lu X.L., Wang W., Han J.J., Xu G.H., Huang Z.X. (2022). Design of a Compact Polarization-Agile and Frequency-Tailored Array Antenna With Digital-Controllable Radiation Beams. IEEE Trans. Antennas Propag..

[11] Cui T.J., Qi M.Q., Wan X., Zhao J., Cheng Q. (2014). Coding metamaterials, digital metamaterials and programmable metamaterials. Light Sci. Appl..

[12] Zhang L., Wang Z.X., Shao R.W., Shen J.L., Chen X.Q., Wan X., Cheng Q., Cui T.J. (2020). Dynamically Realizing Arbitrary Multi-Bit Programmable Phases Using a 2-Bit Time-Domain Coding Metasurface. IEEE Trans. Antennas Propag..

[13] Zhang L., Chen X.Q., Liu S., Zhang Q., Zhao J., Dai J.Y., Bai G.D., Wan X., Cheng Q., Castaldi G. (2018). Space-time-coding digital metasurfaces. Nat. Commun..

[14] Katare K.K., Chandravanshi S., Biswas A., Akhtar M.J. (2019). Realization of Split Beam Antenna Using Transmission-Type Coding Metasurface and Planar Lens. IEEE Trans. Antennas Propag..

[15] Fadhil T.Z., Murad N.A., Rahim M.K.A., Hamid M.R., Nur L.O. (2022). A Beam-Split Metasurface Antenna for 5G Applications. IEEE Access.

[16] Wang M., Ma H.F., Wu L.W., Sun S., Tang W.X., Cui T.J. (2019). Hybrid Digital Coding Metasurface for Independent Control of Propagating Surface and Spatial Waves. Adv. Opt. Mater..

[17] Xu P., Jiang W.X., Cai X., Bai S.H., Cui T.J. (2020). An Integrated Coding-Metasurface-Based Array Antenna. IEEE Trans. Antenas Propag..

[18] (2023). SMP1320-079LF Datasheet. Available via Skywords. https://www.skyworksinc.com/-/media/SkyWorks/Documents/Products/2001-2100/SMPA1320_079LF_203195C.pdf.

[19] Yu N.F., Genevet P., Kats M.A., Aieta F., Tetienne J.P., Capasso F., Gaburro Z. (2011). Light Propagation with Phase Discontinuities: Generalized Laws of Reflection and Refraction. Science.

[20] Bao L., Wu R.Y., Fu X.J., Ma Q., Bai G.D., Mu J., Jiang R.Z., Cui T.J. (2019). Multi-Beam Forming and Controls by Metasurface with Phase and Amplitude Modulations. IEEE Trans. Antennas Propag..

[21] Yang W., Gu L., Che W., Meng Q., Xue Q., Wan C. (2019). A Novel Steerable Dual-Beam Metasurface Antenna Based on Controllable Feeding Mechanism. IEEE Trans. Antennas Propag..

[22] Gu L., Yang W., Xue Q., Che W. (2021). A Dual-Band Steerable Dual-Beam Metasurface Antenna Based on Common Feeding Network. IEEE Trans. Antennas Propag..

[23] Zhang J., Han L., Chen X., Yang R., Zhang W. (2020). Multi-Beam Patch Antenna Based on Metasurface. IEEE Access.

[24] Fan T., Chen X., Han G., Han L., Liu Y., Zhang W. (2021). A multibeam slot antenna using dual-layer metasurface. Electromagnetics.

[25] Li S., Xu F., Wan X., Cui T.J., Jin Y.Q. (2021). Programmable Metasurface Based on Substrate-Integrated Waveguide for Compact Dynamic-Pattern Antenna. IEEE Trans. Antennas Propag..

[26] Lee C.H., Hoang T.V., Chi S.W., Lee S.G., Lee J.H. (2019). Low profile quad-beam circularly polarised antenna using transmissive metasurface. IET Microw. Antennas Propag..

